# Retrospect of the Medical Sciences

**Published:** 1843-06-24

**Authors:** 


### FEVER WITH PERICARDITIS.

At the Sheffield Medical Society, March 23, 1843, Dr. Branson read a paper detailing six cases of fever occurring in one family. The patients were of the respective ages of six, four, eighteen, thirteen, fifteen and nine. The first, a girl, aged six, died ; her case was most interesting on account of i's rarity, from the fact of it being simple idiopathic fever terminating in pericarditis. Dr. Hope mentions no similar case, and Dr. Tweedie observes that "lesions of the heart are very rarely observed in fatal cases of fever." This was, undoubtedly, a case of pure pericarditis supervening upon an attack of fever ; unfortunately, no post-mortem inspection was allowed. Without the aid of the stethoscope the affection of the heart would have been overlooked, as few of the more usual symptoms accompanying pericarditis were present. There was no violent lancinating pain shooting from the heart to the left shoulder and down to the elbow ; no pain on pressing the præcordial region; no bounding palpitation of the organ ; no jerking, hard, and full pulse ; no extended dulness on percussing the region of the heart ; no symptoms of obstructed circulation.

The only marked symptoms were the supine position and a peculiarly anxious and indescribable expression of countenance, indicating the commencement of serious visceral mischief. The stethoscope at once revealed the mystery ; percussion and the stethoscope proved there was no pleuritic inflammation. The absence of valvular murmurs showed that portion of the circulatory system to be uninjured ; and the disappearance of the rubbing sound, without leaving any trace of serous effusion into the pericardium, indicated the case to be one of those to which the term "dry pericarditis" has been given—that is, with effusion of lymph alone, corroborating Bouillaud's axiom, "that the most simple pericarditis is precisely that in which pain is wholly absent, or, at least, but slightly felt." In the treatment four leeches had been applied after some consideration, and the little patient exhibited immediately extreme exhaustion, a blanched countenance and feeble pulse. Reliance was then placed upon digitalis and rapid mercurialisation ; and when the latter took effect the heart symptoms gradually and entirely disappeared, so that for thirteen days previous to death no abnormal sound or irregularity in the circulatory system could be detected. At the commencement of the heart affection the prognosis was most unfavourable ; but when it yielded to the remedies, hopes were entertained. The symptoms which immediately preceded dissolution—the sudden vomiting, the violent abdominal pain, collapsed features, diarrhœa, which ceased when the heart symptoms appeared, and recommenced when they disappeared, small rapid pulse—would seem to indicate that death ensued from intestinal perforation, and not from disease of the heart. From the inquiries made it appeared that this case must have been an isolated one of idiopathic fever ; but there appears to have been a period when, notwithstanding the absence of typhoid symptoms, it had assumed a contagious character. The girl affected with the heart disease was ill from the 21st of August to September 25, when she died. Between September 17 and 20, four of the others were attacked. It seems hardly likely that the latter cases and the first had the same morbific origin ; but it would seem that the disease was communicated to the four on the same day, the latent period slightly varying. Had the first case assumed a typhoid character at the end of the third week, and the other cases followed within ten days, Dr. Branson would have dated the origin of the latter from the commencement of the typhoid symptoms in the former. In this case, though the symptoms usually denominated typhoid never appeared, a morbid change occurred, equally exhausting to nervous energy, and, most probably, equally adapted to the propagation of miasmata.

The variety in the local affections in the cases related was curious ; the heart, brain, nerves, lungs, and bowels, being severally affected in the members of one family, deriving the disease from one common source, forming one exception to the general rule, as stated by Dr. Tweedie, "that when the febrile poison becomes so virulent that every person who has been exposed to its influence has been attacked with fever, the symptoms, in every individual who has received the infection (as if to show the agency of a specific poison,) have been remarkably similar."

To decide the question as to when fever becomes contagious, a great accumulation of facts numerically arranged is required, by which only can a great advance towards checking the fatality of this disorder be made.

In the second case, a girl, aged four, the continued fits of screaming, the almost tetanic spasm of the muscles of the neck, and, at a later period, the contracted pupils and state of coma, seemed to justify an unfavourable prognosis ; yet, recovery was as rapid as it was gratifying. From this case Dr. Branson is inclined to think that too much stress is laid upon the state of the pupil, and quoted an opinion expressed by Dr. H. Holland in his "Medical Notes and Reflections," a most valuable work, in support of this opinion.—*Prov. Med. Journ.*

---

### VESICO-VAGINAL FISTULA.

The "Archives Gènerales de Medecine," for March, contain a very lengthy but interesting communication on the treatment of vesico-vaginal fistula, with the detail of two successful cases, by M. Lallemand, of Montpellier. The first case is that of a lady, twenty-three years of age, labouring under this affliction from the use of instruments in her first labour. The fistula was situated about three and a half inches from the orifice of the urethra ; its transverse diameter was about an inch and a half, the two lips being separated to the extent of half an inch, and the posterior one masked by a fold of the vesical mucous membrane an inch long, one third of an inch high, and one fourth of an inch thick. The greater part of the circumference of the vagina behind the fistula was blocked up and narrowed by semicircular, thick, and hard cicatrices, into which the first phalanx of the index finger could not be passed, whilst the index and medius fingers together could be passed into the bladder through the fistula.

The first step directed by M. Lallemand under these distressing circumstances, was the dilatation of the vagina by large bougies, and afterwards by conical gum elastic cylinders, which unfortunately proved, on the patient's return to Montpellier in two months' time, to have been misapplied, inasmuch as the fistula was much increased in size, and the vaginal contraction in the same state as previously.

M. Lallemand, after having then for a few days attempted to dilate the vaginal passage, on the 22nd of February, 1836, applied the actual cautery on the flap of vesical mucous membrane which blocked up the posterior lip of the fistula, so as completely to destroy it. The sloughs separated in a few days, and the anterior lip having been brought into the same state by the application of the nitrate of silver, the entire edges of the fistula being fresh and bleeding, the hooked catheter (*sonde airigne*) was immediately applied, a task of some difficulty on account of the depth of the fistula and the imposibility of introducing the finger beyond the contraction of the vagina; the vesico-vaginal parietes consequently not being supported, were pierced by the hooks with difficulty and it could not be ascertained whether they were at a proper distance from the posterior lip of the fistula. If they had been too near, the soft parts would have been torn; and if too far, the cervix uteri might have been injured. Guided, however, by the marks engraven on the stem of the instrument, the hooks were confidently forced in, and the instrument being slowly drawn, the fistula could be felt with the index of the other hand to be gradually closing; the movable plate was then wrapped up with lint, and the spring worked until the coaptation was complete, The instrument was then perfectly fixed by its own mechanism. It was withdrawn five days afterwards, and a common elastic catheter passed for a few days, after which the patient made water as usual. Four weeks after the operation, the parts were examined with the speculum, when a transverse band of a bright red colour, half a line wide and more than an inch long, was seen on a level with the contracted part of the vagina, the cicatrices of which prevented the extremities of the band being seen. The patient being now able to retain her water for three or four hours, left Montpellier, to which she returned in the following spring, complaining of fluor albus, the discharge being very liquid. Lallemand at first thought the cicatrix had given way in some part, but this was proved not to be the case by a very careful exploration; there were found two small and tortuous canals on either side of it; the cauterisation of which arrested the discharge. The bladder was kept empty for several days after the application of the actual cautery. The patient recovered perfectly, and the catamenia, which had been absent from the time after confinement, soon reappeared, and resumed their natural course.

In the second case the vesico-vaginal fistula, which was also caused by the use of instruments, was situated about three inches posterior to the meatus urinarius, having a transverse diameter of nearly one inch and a half, and nearly an inch in the antero-posterior direction. The vagina was contracted by two thick cicatrices, but not sufficiently to prevent the passage of the finger beyond the posterior lip of the fistula. The right lower extremity was partially paralytic and contracted. The operation was performed as in the preceding case; the edges of the abnormal communication between the bladder and vagina having been freshened by the actual cautery and the subsequent removal of the sloughs, the parts were brought into coaptation by the hooks of the sonde arigne, which was kept in for several days, and then replaced by a common elastic catheter. The state of the parts was examined a few weeks afterwards with the aid of the speculum, when the fistula was found to be closed, except in the centre, where there was an interval of a line in extent, which soon healed under the application of the nitrate of silver. The patient was dismissed, cured of her infirmity, three months after the performance of the operation.

The proceeding thus advised by Lallemand requires much care in order not to involve any part of the uterus, for a case is recorded by him, in which the hooks having been applied to the part of the bladder covering the womb, and in all probability into the womb itself, pain in the pelvis, speedily extending over the whole abdomen, set in, and notwithstanding the removal of the instrument, symptoms of gastro-enteritis and hepatitis with delirium became manifest, and although the patient for a time survived the attack, she gradually sunk into a state of exhaustion and marasmus, with disturbance of the intellectual functions, and died in the course of the ensuing year. On examination of the body, traces of chronic peritonitis, disease of the liver, and effusion of serum within the cranium were discovered; although the womb was not found to be the seat of organic changes, M. Lallemand is convinced from the symptoms, that it was the source of the complicated diseases which destroyed his patient, and in this opinion he was confirmed by the results of another case which terminated fatally in eight days, the autopsy presenting similar appearances to those in the preceding case. In those cases, therefore, where the fistula is situated on a level with the cervix uteri, it is advisable to attempt a cure only by means of cauterization made with great care, and renewed at long intervals.

The employment of the *sonde airigne* is not adapted for those cases of fistula where the opening is so exceedingly small that it can be closed by the swelling induced by the cauterization of the parts, or where the fistula is of such a size as to occupy the larger part of the vesico-vaginal septum, so that the edges of the fistula could not be brought into contact, or finally where a healthy character of inflammation cannot be excited so as to excite reunion. As a general rule, the chances of success are in the inverse proportion to the duration of the infirmity, because, according to the length of time the diseased state has existed is the degree of irritation and unhealthy inflammation about the parts, and the disordered condition of the general health, consequently the less likelihood is there of a pure and proper character of inflammatory action setting in, and the consequent formation of a firm cicatrix.

It is a task of exceeding difficulty to freshen the entire circumference of the fistula with cutting instruments; some points will escape division, or not be cut with uniformity, while at the same time if a cutting instrument be used effectually, a greater portion of the soft parts is removed, and the opening consequently made larger, without any especial advantage, than when cauterisation is had recourse to. Swelling of the adjacent part is not occasioned, as when the latter process is employed, the tumefaction being of service in rendering the coaptation more easy and more complete. The actual cautery should be had recourse to whenever it is requisite to destroy certain parts and to remove inequalities, especially when the opening is very large, or masked and sinuous, so that its course cannot be followed with a stick of nitrate of silver. The instrument should be of an olivary shape, not more than a line in diameter in the most enlarged part, and the stem still thinner. Probes of different sizes and shapes should be used for the tortuous canals. The inferior paries of the vagina must be protected from the influence of the cautery by the demi-speculum, and the upper part by a large spatula made with an elbow, that there be not any shadow cast by the hand of the assistant. The spatula will serve at the same time to bring the diseased parts into view.

The *sonde-airigne* is preferable to the suture in every case where the fistula is transverse, or can be brought into that form, which can almost always be done when the operation is possible. Out of twenty-one cases, M. Lallemand has only met with one oblique and irregular fistula, the reunion of which could not be effected from behind forwards. If the sutures are not sufficiently tightened, or become relaxed by the slow section of the parts engaged, the lips of the fistula gape, and the operation must be repeated, for there is not any means of increasing the compression. It is not so with the sonde airigne, as the pressure with it can be increased or decreased at pleasure, nor can the edges of the wound get out of place, they being kept *in situ* by the hooks of the instrument, which, at the same time, retain the instrument itself in a state of immobility. It has been employed by M. Lallemand in fifteen cases, and he has been successful with it in nine, not always at the first operation, but at the utmost in two, but the most frequently the application of the caustic has been sufficient to effect a cure afterwards.—*Ibid.*

---

### FIBROUS POLYPUS OF THE UTERUS.

A woman, about forty years of age, pale and exsanguine, was admitted into La Charite under the care M. Velpeau, having laboured under uterine hæmorrhage for four or five years, sometimes to a great extent, at other times more moderately. On examination M. Velpeau discovered the existence of a fibrous polypus springing from the uterus, of such a size that he could not reach the pedicle with his finger. The great state of exhaustion and anæmia of the patient, and the constant hæmorrhage, induced the surgeon to decide on the speedy performance of an operation as the sole chance of saving life, previous to which, however, he drew the attention of the pupils to the case, stating that it could only be mistaken for a cancerous tumour or inversion of the uterus. It would be difficult to make a mistake in a case of cancer, on account of the difference of consistence, the ulceration, and the fœtid discharge, symptoms which would serve to indicate the carcinomatous character of the disease. Inversion of the uterus, however, offers several points of resemblance with fibrous polypi; the anatomical appearance and consistence is the same in both cases, and both give rise to hæmorrhage but in the present instance M. Velpeau stated that this could not be a case of inversion, for the woman had never borne children, and although instances of virgins afflicted with inversion are on record, he doubted the truth of the statements. Besides, an inverted uterus is never of so large a size, and when pressed on with the nail pain is experienced, while the tumour in this case was indolent, and unequal in shape, with prominences just like fibrous polypi; the neck of the uterus could not be reached, whilst in inversion that can be easily effected.

Having decided that the case was one of polypus, and not inversion of the womb, the operation of excision was performed, the tumour being drawn down by long and painful tractions; when it was brought externally to the parts of generation, it had a very long cylindrical pedicle, continuous with the adjacent tissues, but the relative position of the womb could not be ascertained. Velpeau divided what he thought was the pedicle, and then finding there followed some sanguineous discharge, fearing hæmorrhage, he plugged the vagina. The unfortunate sufferer died two days afterwards from peritonitis.

The tumour that had been removed was of a pyriform shape, and about the size of an adult's fist; it was a fibrous substance of a pearly lustre, evidently developed in the tissue of the uterus, and covered with a very thin layer of the uterine parenchyma. At the autopsy Velpeau showed the pupils the cylinder which came away with the polpus, and which bore some resemblance to the hypertrophied cervix uteri, but a careful examination showed that the ovaries and round ligaments were contained in a species of funnel, composed of the inverted body of the womb.—*Prov. Med. Journ.*, *from Journ des Connais. Med. Chir.*, March, 1843.

---

### *On Electro-puncture in the Treatment of Deafness, depending on a Paralysis of the Acoustic Nerve.* By M. Jobert.

The paralysis of the acoustic nerve may be produced by exposure to a current of air, to too great a shock of the head, to waves of sound too violent, to affections of the teeth or of the gums. Electropuncture has been already employed in these cases, but it had fallen into disrepute. The author believes that he uses it in a manner more direct and more rational; here is his proceeding:—Stard's sound, he says, is introduced through the nasal fossa into the eustachian tube, and in this sound a long thin acupuncture needle is inserted, so as to fix itself in a point of the parietes of the eustachian tube, while the other end projects from the end of the sound; another acupuncture needle is implanted in the membrane of the tympanum. This being done, one of the conducting wires of a galvanic battery, of which the trough is filled with water and muriatic acid, is passed through the eye of one of the needles, and the end of the other conducting wire is made to touch the opposite needle. I have used, in the beginning, eight parts of the battery, then I got to ten, to twelve pairs; finally, I have been as high as eighteen, and at present I have patients who have undergone several sittings, and on whom I have acted with the entire pile, the trough of which contains forty metallic pairs. At the moment that the two poles are put into contact, there is a very painful shock in the ear and in the head, with convulsive motions; but this shock and this pain cease immediately. In a single patient the impression was felt during eight days, but it never extended beyond a slight pain, which ceased of itself. It must be added, that the patients who were submitted to electricity in this manner, were, during some moments, as if stunned, and preserved some time after the experiment a bewildered look. The sitting was usually confined to a single shock when the patients were irritable; I have given two, and even three shocks, in people whose sensibility was obtuse, and who have been already submitted to electro-puncture. In general I allow eight days to pass between each trial. The author then relates four cases of well marked deafness, and in which the cure was complete; in the first after a single shock, in the second after two shocks, and in the third after two sittings, each composed of three galvanic shocks.—*Dublin Journal of Medical Science*, May, 1843, *from L'Examinateur Medicale.*

